# Emotional and behavioral problems in Japanese preschool children with subthreshold autistic traits: findings from a community-based sample

**DOI:** 10.1186/s12888-022-04145-1

**Published:** 2022-07-26

**Authors:** Aya Shirama, Andrew Stickley, Yoko Kamio, Aya Saito, Hideyuki Haraguchi, Ayumu Wada, Kazuki Sueyoshi, Tomiki Sumiyoshi

**Affiliations:** 1 Department of Preventive Intervention for Psychiatric Disorders, National Institute of Mental Health, National Center of Neurology and Psychiatry, 4-1-1 Ogawahigashicho, Kodaira, Tokyo, 187-8553 Japan; 2 Institute of Education and Human Development, Ochanomizu University, 2-1-1 Otsuka, Bunkyo-ku, Tokyo, 112-8610 Japan; 3 Human Science Division, Faculty of Core Research, Ochanomizu University, 2-1-1 Otsuka, Bunkyo-ku, Tokyo, 112-8610 Japan

**Keywords:** Subthreshold autistic traits, Emotional and behavioral problems, Preschool children, Cognitive function

## Abstract

**Background:**

Recently, there has been a growing recognition that autistic traits exist along a continuum beyond diagnostic categories and that even subclinical symptoms may be associated with an increased risk for the psychosocial well-being and mental health of children. However, as yet, there has been little research on whether preschool children with autism spectrum disorder (ASD) symptoms, who do not meet the diagnostic criteria for ASD, are more likely to experience difficulties. To address this deficit this study examined whether young children with subthreshold autistic traits have an increased risk for emotional/behavioral difficulties.

**Methods:**

Data were analyzed from 1057 Japanese preschool children aged 5-years old collected during the first wave of the Tama Children’s Survey (TCS) cohort study. Parent-reported autistic traits were assessed with the Social Responsiveness Scale (SRS), while they provided information on their child’s emotional/behavioral problems using the Strengths and Difficulties Questionnaire (SDQ). Logistic regression analysis was used to examine associations.

**Results:**

Preschool children with mild-to-moderate autistic traits, corresponding to subclinical autism were significantly more likely to score above the clinical thresholds for emotional/behavioral problems compared to children with fewer autistic traits. Follow-up diagnostic assessments and analyses of 72 children from the cohort confirmed these findings and showed that these children with subthreshold autistic traits also had a significantly lower intelligence quotient (IQ) as measured by the Wechsler Preschool and Primary Scale of Intelligence (WPPSI).

**Conclusions:**

Although subthreshold autistic traits are difficult to define due to the sometimes vague border between typical and atypical development, there may be a large number of preschool children with subthreshold autistic traits, who may have an increased risk for a variety of different emotional/behavioral difficulties as well as lower cognitive functioning.

## Background

Autism spectrum disorder (ASD) is a neurodevelopmental disorder that is diagnosed based on key symptoms including impaired social communication and interpersonal relationships as well as restricted interests and repetitive behaviors [1]. Following Kanner’s first report of cases of autism [2], ASD was traditionally considered as a distinct clinical condition. However, in more recent decades research has emerged which has questioned this distinction. For example, it has been shown that parents of children with ASD often exhibit milder forms of autistic traits referred to as the broader autism phenotype (BAP) [3], while subsequently, in a large cohort sample of siblings at familial high-risk of ASD, the BAP was detected in high-risk siblings who did not have ASD by age 3 years [4]. This research has culminated in a growing body of evidence being amassed from different countries (e.g. the USA, the Netherlands, and Japan), which suggests that autistic traits are continuously distributed in the general population [5–7]. Autistic traits can be measured quantitatively as a broad manifestation of the autism phenotype that includes social impairments, communication impairments, and restricted and repetitive behaviors and interests [8], with general population twin studies showing that similar heritability and risk factors underlie autistic traits and ASD [5, 9, 10]. Given the increasing realization that ASD is not a discrete disorder and that ASD may represent the extreme end of an autistic traits continuum in the general population (see [11] for a review), it is now assumed that there might be a larger number of children who have mild-to-moderate autistic traits that correspond to subthreshold conditions [12, 13] compared to children with ASD considering the continuous nature of this distribution. In connection with this, while many previous studies have reported the negative impact of ASD on mental health (e.g., [14–16]), as yet, little research has focused on subthreshold autistic traits in this context as they usually remain undiagnosed in the framework of DSM criteria against a backdrop where autism is understood as being characterized by intellectual and language disabilities as well as severe impairment in social interactions [17].

Subthreshold autistic traits have been found to influence the psychosocial well-being and mental health of both clinical and community-based children. For example, data from the Avon Longitudinal Study of Parents and Children (ALSPAC) indicate that social-communicative deficits, assessed by the parent-reported Social and Communication Disorders Checklist (SCDC. [18]), were significantly associated with behavioral problems, assessed by the teacher-reported Strengths and Difficulties Questionnaire (SDQ), in community-dwelling children aged 7–8 years old, even after controlling for sex, IQ and the mother’s educational level [19]. Support for the potential connection between subthreshold autistic traits and poorer psychosocial well-being also comes from research that has reported elevated levels of autistic traits in child and adolescent patients with anxiety disorders and/or mood disorders [20, 21]. A recent psychopathological model assumes subthreshold autistic traits as a common vulnerability factor for a wide variety of mental disorders including anxiety and mood disorders, schizophrenia, trauma and stress-related disorders [22, 23]. Thus, the risk for mental health problems in children with subthreshold autistic traits should be clarified based on epidemiological or clinical evidence.

Several twin and cross-sectional studies have also investigated whether different levels of autistic traits have a negative impact on psychosocial functioning in childhood [12–14]. Lundström et al. (2011) classified 11,222 children aged 9 and 12-years-old into six groups ranging from low-risk cases to probable ASD cases (corresponding to a clinical level) based on autistic trait scores from the Autism-Tics, ADHD (attention deficit hyperactivity disorder) and other Co-morbidities inventory (A-TAC [24]). They found that children with subthreshold autistic traits, comprising 28% of the entire sample, had an increased risk for ADHD and anxiety symptoms as well as conduct problems [7]. In another study that used data from the general population in elementary and junior high schools (*N* = 24,728), subthreshold autistic traits were associated with emotional and behavioral problems [13]. More specifically, Moriwaki and Kamio (2013) divided their sample into three categories–low-risk, medium-risk, and high-risk–based on their autistic traits scores as measured by the Social Responsiveness Scale (SRS, [25]). They found that compared to children in the low-risk group, children in the medium-risk group (12% of the sample) had an increased risk of emotional and conduct problems as assessed by the SDQ, although the odds for problems were greatest in the high-risk group (2.4% of the sample) [13]. Overall, data from the above-mentioned studies indicate that the proportion of children with subthreshold autistic traits may far exceed the number diagnosed with ASD (i.e. 1.4 to 2.64% [26, 27]), and that subthreshold autistic traits may be associated with psychiatric problems in childhood.

Despite the evidence discussed above, relatively little is known about the relationship between subthreshold autistic traits and emotional/behavioral problems in preschool children. For example, Möricke et al. (2010) reported that subclinical autistic traits at age 14–15 months, measured by the Early Screening of Autistic Traits Questionnaire (ESAT [28, 29]), were associated with behavioral and cognitive problems at age 3–5 years [12], assessed with the Child Behavior Checklist [30]. However, there is variability in autistic traits between 14 and 15 months and 4–5 years of age [12], and some early autism screening measures may have difficulties distinguishing autistic traits from other aspects of abnormal development [29]. Also, a recent study reported that the diagnostic stability of autism increases as preschool children age [31]. These considerations suggest a need for research that uses different forms of assessment, and focuses on older children.

Thus, in order to examine the hypothesis that subclinical autistic traits are associated with an increased risk for psychosocial problems in preschool children, this study analyzed two sets of data. First, we used screening data from a large population-based (*N* = 1057) cohort of preschool children aged 5 years to determine the association between the severity of autistic traits and emotional/behavioral problems. Second, in order to obtain detailed diagnostic information, 72 children from the cohort aged 5- to 6-years old also took part in a follow-up assessment and underwent a clinical interview. We decided to use two different assessment criteria i.e. parent-reported autistic traits and diagnostic assessments to examine associations given that autistic traits are distributed continuously and there is no clear point of discontinuity, while the range of subthreshold autistic traits can vary depending on the categorization criteria used. As comparatively little is known about the association between subclinical autistic traits and psychosocial problems in preschool children, this study will elucidate this association while further expanding the knowledge base about the effects of subclinical autistic traits in young children.

## Methods

### Participants and procedure

The data used in this study came from the Tama Children’s Survey (TCS). The TCS is a prospective population-based cohort study investigating the associations between autistic symptoms, mental health, and the social development of children living in the Tama district of Tokyo, Japan. In Wave 1 of the study in 2012, 2953 families with a child in kindergarten and nursery school classes for 5-year-olds were approached to take part in the study. Valid responses were obtained from 1406 families (a response rate of 47.6%). Further details of the study recruitment, retention, and data collection procedures have been described previously elsewhere [32–35]. Data were collected on a variety of developmental traits as well as sociodemographic factors using a postal questionnaire that was self-completed by parents. Ethical approval for the study was obtained from the Ethics Committee of the National Center of Neurology and Psychiatry, Japan. All procedures were carried out in accordance with relevant guidelines and regulations. Informed consent was obtained from parents/legal guardians. Before commencement of the research the purpose and ethical/legal policies of the study were explained to the parents who were then asked to answer and return the questionnaire if they agreed to participate in the study. In the present study, data were analyzed from 1057 Wave 1 children (565 boys) who had no missing information for autistic traits and behavioral and emotional problems.

**Fig. 1:**
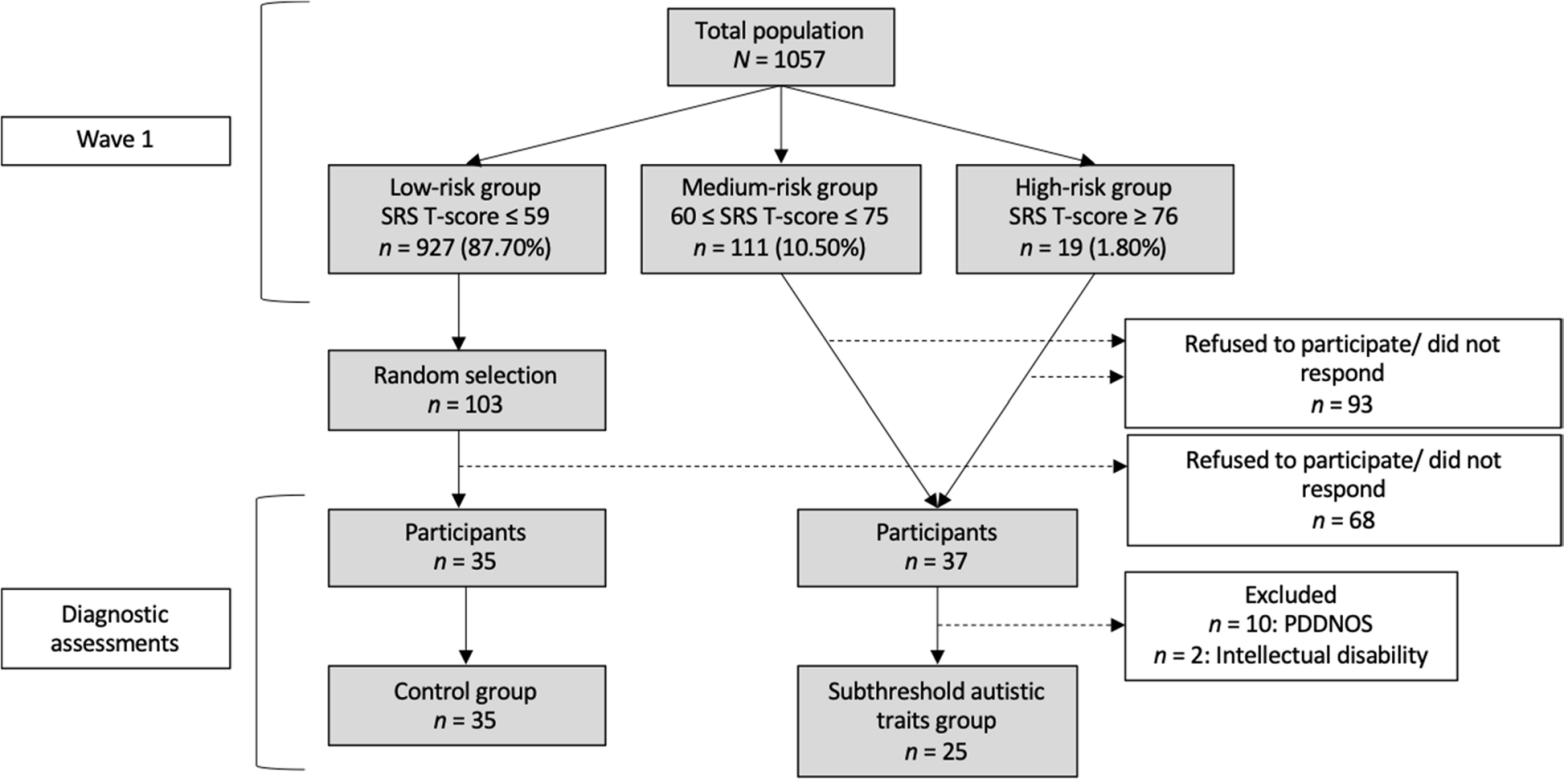
Flow Chart of Participants

We also invited 184 families to take part in an additional diagnostic assessment. This group consisted of (i) 103 randomly selected families with children in the Low-risk group for autistic symptoms (see below for further details), (ii) all the families of children in the Medium-risk group, and (iii) all the families of children in the High-risk group. However, refusals and the non-response of some families meant that the diagnostic assessment included children and parents from 72 families (39.13% of the 184 invited families): 42.11% of the children with the highest autistic traits scores (*n* = 8, 6 boys, 2 girls), 26.13% with mild-moderate autistic traits scores (*n* = 29, 19 boys, 10 girls), and 33.98% with low autistic traits scores (*n* = 35, 17 boys, 18 girls). A flow chart of the participants is presented in Fig. 1.

### Measures

#### Autistic traits (independent variable)

Autistic traits in the children at age 5 years were assessed with the Japanese version of the Social Responsiveness Scale (SRS [25, 36];). Detailed information about the process of developing the Japanese version of the SRS was reported in an earlier research article [37]. The SRS is a 65-item quantitative measure of autistic traits for children aged 4–18 years old. Previous epidemiological studies have reported that SRS scores have a normal distribution in the Japanese population [6, 38]. In the current study, we used the original criteria proposed by the scale’s developers [25] to divide the SRS scores of the children into three groups with different thresholds: (i) children with the highest autistic traits, almost corresponding to clinical level ASD (the High-risk group, with a T-score ≥ 76, *n* = 19 (1.80%)), (ii) children with mild-moderate autistic traits almost corresponding to subthreshold ASD (the Medium-risk group, with a T-score ≥ 60 and ≤ 75, *n* = 111 (10.50%)), (iii) children with a low risk for autistic traits (the Low-risk group, with a T-score ≤ 59, *n* = 927 (87.70%)). Based on the Japanese norms reported in an earlier study, T-scores with a cutoff of 60, and 75 correspond to approximately 15, and 2% of the sex-specific distribution, respectively [38]. The SRS has been shown to have a two-factor structure (with 1 factor comprising 53 social communication interaction items [39], while the other comprises 12 restricted and repetitive behavior [RRB] items) that corresponds to DSM-5 criteria [40]. Cronbach’s alpha for the total score was 0.90 in this study and ranged from 0.77 (restricted/repetitive behavior) to 0.83 (social communication/interaction) for the subscale scores, indicating good internal reliability. A longitudinal study of school-aged twins found that individual differences in SRS scores were highly preserved during a 5-year follow-up period [41]. Recently, both lower and higher autistic traits assessed by the SRS have been shown to remain stable from 5 to 8 years of age [32].

#### Emotional and behavioral problems (dependent variable)

To assess the extent of emotional and behavioral problems, the SDQ [42] was completed by parents. The SDQ is a standardized screening questionnaire consisting of 25 items that measure emotional and behavioral problems in children and adolescents aged 3–16 years old [43, 44]. Each item is scored on a scale ranging from 0 ‘not true’, 1 ‘somewhat true’, to 2 ‘certainly true’. The SDQ has five subscales: emotional symptoms, conduct problems, hyperactivity-inattention, peer problems, and prosocial behavior. When combined, four of the subscale scores (excluding prosocial behavior) yield a total difficulties score. Following the test developer’s guidelines, the 90th percentile was used as a cutoff across all subscales to predict a substantially raised probability of diagnosed psychiatric disorders [45]. The validity and reliability of the Japanese version of the SDQ have been demonstrated in previous studies [46, 47]. In the current study, Cronbach’s alphas ranged between 0.53 (peer problems) to 0.73 (hyperactivity-inattention) for the subscale scores and 0.81 for the total difficulties score. This range of values accords with those of previous studies that have used the SDQ in school-aged children and adolescents [46, 48].

#### Covariates

Control variables were chosen that have been linked to emotional and behavioral problems in children aged 5- to 7-years old in previous studies [43, 44]. The child-related variables included: gender, age, number of siblings. The parent and family variables were the length of the mother’s education (in years) and family income (in millions of Yen). Previous studies have shown that socioeconomic factors including family income and the mother’s education can affect many aspects of children’s development and result in mental health and behavioral problems, lower academic achievement, and obesity [49, 50]. We also obtained information about the mother’s depressive symptoms which have been linked to children’s emotional and behavioral problems in numerous studies and reviews (e.g., [51]). For example, a previous study has shown that mothers of children with ASD experience high levels of stress that have been associated with an increased risk for the current treatment of maternal depression [52]. We obtained information about the mother’s depressive symptoms using the case-finding instrument tool (TQI) which was extracted from the Primary Care Evaluation of Mental Disorders questionnaire [53]. The TQI consists of two questions about depression and anhedonia: (1) ‘During the past month, have you often been bothered by feeling down, depressed, or hopeless?’ and (2) ‘During the past month, have you often been bothered by little interest or pleasure in doing things?’ Answers are combined to generate a continuous score that ranges from 0 to 2 with higher scores indicating greater depression. Adachi et al. have previously demonstrated the utility of this measure for assessing depressive symptoms in Japanese adults [54].

### Diagnostic assessments

In order to gain further insight into the association between higher autistic traits and emotional/behavioral problems in children that had been assessed for autistic traits with a screening instrument, we invited 184 children and their parents from the Low- to High-risk groups in Wave 1 to undergo further assessment. Among them, 72 families participated in a further diagnostic assessment. Specifically, 37 children in the Medium- and High-risk groups underwent a diagnostic assessment where autistic traits and intellectual abilities at age 5- to 6-years old were assessed by experienced clinicians (clinical psychologists) using the Autism Diagnostic Interview-Revised (ADI-R) [55] and Autism Diagnostic Observation Schedule (ADOS) [56]. Children’s intellectual ability was assessed with the Japanese version of the Wechsler Preschool and Primary Scale of Intelligence (WPPSI [57]). Impaired cognitive functioning is one of the most common co-occurring features in ASD [58, 59]. Despite this, until now there has been little research that has examined the link between subthreshold autistic traits and cognitive functioning in young children even though two previous population-based sample studies have indicated that subclinical autistic traits may be associated with problems in cognitive functioning [12, 60].

A child psychiatrist also obtained information from the parents about their child’s medical history including the family history of mental illness, concerns about their child’s development, developmental history, and mental/physical impairments. Additionally, parents were interviewed about their children’s lifetime (present and past) psychiatric problems including anxiety disorders and obsessive-compulsive disorder using the Schedule for Affective Disorders and Schizophrenia for School-Age Children Present and Lifetime version (K-SADS-PL) [61]. To generate a clinical diagnosis, all relevant data, including the ADOS, ADI-R, and WPPSI scores, as well as the child’s medical history were reviewed by a clinical team that consisted of a child psychiatrist and clinical psychologists. Confirmed diagnoses met DSM-IV-TR criteria [62] and resulted in 10 children being classified as having pervasive developmental disorder not otherwise specified (PDDNOS). None of the children were diagnosed as having autistic disorder or Asperger’s disorder. The remaining 27 children were classified as being without an ASD diagnosis but as having higher SRS scores. Two of these children were subsequently excluded due to mild-moderate intellectual impairment. Finally, 25 children (aged 67 to 82 months) were categorized as belonging to the subthreshold autistic traits group.

Thirty-five children from the Low-risk group in Wave 1 (aged 60 to 76 months) served as the control group in the follow-up assessment. Children’s intellectual abilities were assessed with the WPPSI. A child psychiatrist also obtained clinical information from the parents. None of the children were diagnosed as having an intellectual disability or ASD.

### Analysis

Descriptive statistics for the Low-risk, Medium-risk, and High-risk cases (the subthreshold autistic traits group and the control group in the diagnostic assessment) were calculated and then compared using either one-way analysis of variance (ANOVA) or Chi-square tests as appropriate. Hierarchical logistic regression analysis was then used to assess emotional and behavioral problems in children with Medium−/High-risk autistic traits compared to children with Low-risk autistic traits. Each emotional/behavioral outcome was assessed in a separate analysis with the use of three models. In Model 1 the bivariate association between the Medium−/High-risk groups and each emotional/behavioral problem was examined. We then assessed the role of child and parent/family covariates (Model 2) and the mother’s mental health (Model 3) in the association between autistic traits and emotional/behavioral outcomes. The data from the diagnostic assessments, i.e. descriptive statistics of the subthreshold autistic traits group and the control group were compared using either one-way ANOVA tests with age as a covariate or Chi-square tests as appropriate. All analyses were performed with IBM SPSS Statistics version 23. Results are presented as odds ratios (OR) with 95% confidence intervals (CI). The level of statistical significance was set at *p* < 0.05 (two-tailed).

## Results

### Sample characteristics

Descriptive statistics for the Low-risk, Medium-risk, and High-risk groups are presented in Table 1. In the Low-risk group there were a larger number of siblings, more mothers were highly educated, while the mother’s depressive symptoms were lower compared to in the other groups. There were no differences between the groups with regard to age, the male-to-female ratio, or family income. The Medium-risk and High-risk groups were more likely to have significantly higher SDQ scores than the Low-risk group.

**Table 1:** Sample Characteristics of the Low-, Medium-, and High-Risk Groups (*N* = 1057)

	Low-risk group	Medium-risk group	High-risk group	*F/x*^*2*^*, p*
	*n* = 927	*n* = 111	*n* = 19	
Child characteristics				
Age in years, mean (SD)	5.34 (0.34)	5.29 (0.31)	5.27 (0.29)	*F* (2,1055) = 1.55, *p* = .21
Male, number (%)	502 (54.15)	51 (45.95)	12 (63.16)	*x*^2^ = 3.56, *p* = .17
Siblings, number (SD)	2.06 (0.73)	1.87 (0.73)	1.79 (0.63)	*F* (2,1055) = 4.37, *p* < .01^a^
Parent/family characteristics				
Education mother				
University/higher professional (%)	85.68	73.33	78.95	*x*^2^ = 9.31, *p* = .010^c^
Family income (million yen) (%)				
Under 2	1.77	4.12	0	*x*^2^ = 11.53, *p* = .31
2 or more and less than 5	21.32	28.87	16.67	
5 or more and less than 7	30.98	34.02	33.33	
7 or more and less than 10	30.51	23.71	44.44	
10 or more and less than 15	13.19	8.25	5.56	
15 or more	2.24	1.03	0	
Mother’s depression (number of “yes” answers) (%)				
0	75.25	51.85	42.11	*x*^2^ = 38.22, *p* = .0001^c^
1	15.12	25.93	26.32	
2	9.64	22.22	31.58	
Autistic traits (SRS), mean (SD)				
Social communication/interaction	25.35 (8.18)	47.66 (5.88)	69.79 (11.25)	*F*(2,1055) = 634.55, *p* < .001^a,b^
Restricted/ repetitive behavior	3.46 (2.55)	9.41 (3.29)	17.74 (4.79)	*F*(2,1055) = 481.47, *p* < .001^a,b^
Total raw score	28.81 (9.72)	57.07 (7.29)	87.53 (14.49)	*F*(2,1055) = 738.79, *p* < .001^a,b^
Emotional/behavioral problems (SDQ), mean (SD)				
Emotional symptoms	1.27 (1.43)	2.89 (2.20)	3.90 (3.02)	*F*(2,1055) = 75.13, *p* < .001^a,b^
Conduct problems	1.68 (1.42)	3.14 (1.82)	3.42 (2.36)	*F*(2,1055) = 57.32, *p* < .001^a,b^
Hyperactivity-Inattention problems	2.63 (1.92)	4.74 (2.16)	6.21 (2.52)	*F*(2,1055) = 83.95, *p* < .001^a,b^
Peer problems	1.02 (1.12)	2.62 (1.83)	4.73 (1.28)	*F*(2,1055) = 748.49, *p* < .001^a,b^
Prosocial behavior	7.13 (1.89)	5.96 (2.03)	4.00 (2.67)	*F*(2,1055) = 40.67, *p* < .001^a,b^
Total difficulties	6.59 (3.92)	13.39 (4.84)	18.26 (5.65)	*F*(2,1055) = 203.90, *p* < .001^a,b^

*SD* Standard deviation, *SRS* Social Responsiveness Scale, *SDQ* Strengths and Difficulties Questionnaire

^a^ Post-hoc tests with an ANOVA showed a significant difference between the Low-risk group and the Medium-risk group (p < .05)

^b^ Post-hoc tests with an ANOVA showed a significant difference between the Low-risk group and the High-risk group (*p* < .05)

^c^ Residual analyses with a Chi-square test showed a significant difference between the Low-risk group and the other groups (p < .05)

### Autistic traits and emotional and behavioral difficulties

The results from the logistic regression analysis examining the association between the level of autistic traits and emotional and behavioral difficulties are presented in Table 2. Compared to the Low-risk group, the Medium-risk group had significantly increased odds for all outcomes that ranged from 3.80 (95% confidence interval [CI]: 2.23–6.47) for reduced prosocial behavior to 25.18 (95% CI; 13.43–47.20) for overall difficulties (total score) in the bivariate model (Model 1). Although adjustment for child and parent/family variables (Model 2) and mother’s depression (Model 3) led to a reduction in the odds ratios for emotional symptoms, conduct problems, hyperactivity/inattention and the total symptoms score, the Medium-risk group continued to have significantly increased odds for all outcomes with ORs ranging from 3.86 (95% CI: 2.19–6.81) for reduced prosocial behavior to 26.90 (95% CI: 10.40–69.62) for peer problems. In the fully adjusted Model 3, the High-risk group had higher odds than the Low-risk group for all outcomes that ranged from 11.16 (95%CI: 3.86–32.31) for conduct problems to 241.79 (95% CI: 66.90–873.83) for peer problems.

**Table 2:** Association between the Level of Autistic Traits and Emotional and Behavioral Problems in Preschool Children

		Model 1	Model 2	Model 3
		OR (95% CI)	OR (95% CI)	OR (95% CI)
Emotional symptoms	Low-risk group	Ref.	Ref.	Ref.
	Medium-risk group	6.79 (3.74–12.34)****	5.88 (3.19–10.83)****	4.86 (2.60–9.10)****
	High-risk group	15.84 (5.84–43.01)****	15.78 (5.60–44.46)****	11.97 (4.11–34.90)****
Conduct problems	Low-risk group	Ref.	Ref.	Ref.
	Medium-risk group	6.92 (3.89–12.31)****	7.40 (4.06–13.50)****	5.95 (3.21–11.03)****
	High-risk group	14.40 (5.33–38.86)****	14.83 (5.34–41.18) ****	11.16 (3.86–32.31)****
Hyperactivity/ inattention	Low-risk group	Ref.	Ref.	Ref.
	Medium-risk group	7.31 (4.13–12.93)****	7.62 (4.14–14.02)****	6.74 (3.63–12.52)****
	High-risk group	22.21 (8.47–58.21)****	21.51 (7.80–59.28)****	18.41 (6.55–51.71)****
Peer problems	Low-risk group	Ref.	Ref.	Ref.
	Medium-risk group	23.90 (9.65–59.20)****	26.98 (10.63–68.47)****	26.90 (10.40–69.62)****
	High-risk group	212.08 (64.36–698.89)****	242.78 (69–851.89)****	241.79 (66.90–873.83)****
Reduced prosocial behavior	Low-risk group	Ref.	Ref.	Ref.
	Medium-risk group	3.80 (2.23–6.47)****	3.76 (2.16–6.54)****	3.86 (2.19–6.81)****
	High-risk group	12.20 (4.77–31.16)****	11.39 (4.34–29.90)****	11.82 (4.43–31.52)****
Total difficulty score	Low-risk group	Ref.	Ref.	Ref.
	Medium-risk group	25.18 (13.43–47.20)****	25.54 (13.29–49.08)****	21.68 (11.18–42.03)****
	High-risk group	86.32 (30.25–246.30)****	83.36 (28.57–243.22)****	68.56 (23.06–203.89)****

*OR* Odds ratio, *CI* Confidence interval, *Ref* Reference category

Model 1: Bivariate association between the three severity classes of autistic traits and emotional and behavioral problems

Model 2: Adjusted for gender, age (in years and months), siblings (number), mother’s education (in years), family income (in millions of Yen)

Model 3: Adjusted for the variables in Model 2 and mother’s depression (continuous score from 0 to 2)

**** *p* < .0001, *** *p* < .001, **p < .01 * < .05 (two-tailed)

### Comparison between control and subthreshold autistic children who participated in the diagnostic assessment

Descriptive statistics for child characteristics, autistic traits, emotional and behavioral outcomes and for intelligence quotient for the control and subthreshold autistic children who participated in the diagnostic assessment are presented in Table 3. There were no differences between the groups in the male-to-female ratio. The subthreshold autistic children were significantly younger than the children in the control group. Children with subthreshold autistic traits had significantly higher scores for all of the SDQ subscales. Regarding intelligence, the subthreshold autistic traits group had significantly lower WPPSI scores for all outcomes. We also found that 16% of the subthreshold autistic traits group were diagnosed as having anxiety disorders (specific phobia: 1, social phobia: 2, separation anxiety disorder and obsessive-compulsive disorder: 1 (data not tabulated)).

**Table 3:** Comparing Control and Subthreshold Autistic Traits Children Who Participated in the Diagnostic Assessment

	Control	Subthreshold ASD	*F/x*^*2*^*, p*
	n = 35	*n* = 25	
Child characteristics			
Age in years, mean (SD)	6.19 (0.36)	5.76 (0.31)****	*F*(1,59) = 23.56, p < .0001
Male, n (%)	18 (51.43)	17 (68.00)	x^2^ = 2.24, *p* = .13
Autistic traits (SRS), mean (SD)			
Social communication/interaction	24.51 (10.47)	48.09 (10.07)****	*F*(1,59) = 122.53, *p* < .0001
Restricted/ repetitive behavior	3.67 (3.64)	10.40 (4.32)****	*F*(1,59) = 61.58, *p* < .0001
Total raw score	28.18 (13.63)	58.49 (12.66)****	*F*(1,59) = 127.71, *p* < .0001
Emotional/behavioral problems (SDQ), mean (SD)			
Emotional symptoms	1.23 (1.31)	3.08 (2.24)****	*F*(1,59) = 14.83, *p* < .0001
Conduct problems	1.37 (1.19)	3.46 (1.77)****	*F*(1,59) = 25.96, *p* < .0001
Hyperactivity/Inattention problems	1.91 (1.48)	5.25 (2.56)****	*F*(1,59) = 35.36, *p* < .0001
Peer problems	1.03 (1.34)	3.00 (1.79)****	*F*(1,59) = 27.25, *p* < .0001
Prosocial behavior	7.11 (1.89)	5.29 (2.22)****	*F*(1,59) = 16.01, *p* < .0001
Total difficulties	5.54 (3.11)	14.79 (5.18)****	*F*(1,59) = 69.28, *p* < .0001
Intelligence quotient (WIPSSI), mean (SD)			
VIQ	109.64 (15.78)	94.75 (17.50)**	*F*(1,59) = 8.66, *p* < .005
PIQ	118.94 (15.36)	107.25 (12.70)*	*F*(1,59) = 5.91, *p* < .05
FIQ	117.00 (15.89)	100.60 (14.37)**	*F*(1,59) = 8.78, *p* < .005

Univariate analyses of variance with age as a covariate were used for continuous data and Chi-square analyses for categorical variables

The significance results refer to Control vs. Subthreshold ASD

*SD* Standard deviation, *SRS* Social Responsiveness Scale, *SDQ* Strengths and Difficulties Questionnaire, *WPPSI* Wechsler Preschool and Primary Scale of Intelligence, *VIQ* Verbal Intelligence Quotient, *PIQ* Pictorial Intelligence Quotient, *FIQ* Full Intelligence Quotient

The SRS and SDQ scores were measured in Wave 1

**** *p* < .0001, *** p < .001, **p < .01 * < .05

We also examined differences in terms of sociodemographic factors, and the SRS and SDQ scores between the 37 Medium−/High-risk children who participated in the diagnostic assessment and the 93 children who were invited but did not participate in the assessments. The results from independent *t* tests (or Chi-square tests) showed that there were no differences between the groups for age (*t*(128) = 0.31, *p* = .76), mother’s education (*x*^2^ = 11.55, *p* = .40), or family income (*x*^2^ = 4.00, *p* = .78). There were also no differences for four of the SDQ measures (emotional symptoms *t*(128) = − 0.42, *p* = 0.68, conduct problems: *t*(128) = 0.70, *p* = .49, hyperactivity/inattention: *t*(128) = − 1.20, *p* = .23, and the total difficulties score: *t*(128) = − 1.36, *p* = 0.18), or for the SRS measures (RRB: *t*(128) = − 1.61, *p* = .11, SC: *t*(128) = − 0.64, *p* = .52, Total score: *t*(128) = − 1.01, *p* = .31). However, children who participated in the diagnostic assessments had higher scores for SDQ peer problems (*t*(128) = − 2.48, *p* < .05) and lower SDQ prosocial behavior scores (*t*(128) = 2.27, *p* < .05) (data not tabulated).

## Discussion

The aim of this study was to examine the association between subthreshold autistic traits and emotional and behavioral problems in community-based preschool children (mean age: 5.30 years). We hypothesized that even subclinical autistic traits might be associated with psychosocial problems in early childhood. Results from logistic regression analyses of data from 1057 children collected during the TCS showed that children in the Medium-risk group for autism had significantly increased odds for all SDQ domains after adjusting for child, parent/family, and mother’s mental health variables compared to children in the Low-risk group. Although children in the Medium-risk group had lower odds for behavioral/emotional outcomes compared to those in the High-risk group, they were significantly more likely to score above the clinical threshold on all of the SDQ emotional/behavioral domains compared to children in the Low-risk group.

The results of this study are consistent with earlier findings from community-based/twin studies of older (school-aged) children, which reported that subthreshold autistic traits increase the risk for mental health problems [7, 13]. These studies categorized 12–28% of children with mild-to-moderate autistic traits as having subthreshold autistic traits, and showed that this proportion far exceeds the number diagnosed with ASD. In our study 111/1057 (10.50%) children were classified as belonging to the Medium-risk group, with subthreshold autistic traits based on the original categorization criteria proposed by the SRS developers [25, 36]. Together with earlier results, the findings from the current study suggest that there may be a large number of both preschool- and school-aged children with subthreshold autistic traits, who may have an increased risk for a variety of different emotional/behavioral difficulties.

Previous research has shown that children with ASD often experience emotional difficulties including heightened levels of anxiety and depression [63, 64]. As regards autistic traits, a community-based twin study (3233 twins) of children aged 8–9 years reported a modest correlation between autistic traits and internalizing traits including fear, generalized anxiety, and social anxiety [65], while other longitudinal research that used a general population sample of preschool/school children found that autistic traits predicted later SDQ emotional symptom scores [33, 66]. The present results accord with this previous research by showing that preschool children with mild-to-moderate autistic traits (the Medium-risk group in Wave 1) were significantly more likely to score above the clinical threshold for emotional problems. Moreover, the diagnostic assessment showed that 4 of 25 children in the subthreshold autistic traits group had a clinical level of anxiety disorder symptoms. Indeed, the fact that some research has highlighted that children and adolescents with anxiety disorders and/or mood disorders also exhibit higher scores on ASD symptom scales than healthy controls [20, 21], suggests that autistic traits and emotional difficulties/psychopathology might be closely connected in some children, with genetic factors possibly underlying both phenomena [67].

Children in this study with mild-to-moderate autistic traits also had an increased risk for conduct problems, which are known to be frequent in children with ASD. For example, the results from a study using a large clinical sample (*N* = 400) showed that one in four children aged 2–16.9 years had a score on the aggressive behavior scale of the Child Behavior Checklist (CBCL) in the clinical range [68]. Our results are in line with those from a previous study, where subthreshold autistic traits were associated with an increased risk for aggressive behavior as measured by the CBCL at age 3 [12]. In addition, as shown in Table 2, the odds ratio for conduct problems in children with autistic traits decreased when maternal depression was adjusted for in the analysis. This indicates that maternal depression is associated with conduct problems in the Mid−/High-risk group. Given that conduct problems have been shown to be predictors of caregiver stress in children with ASD [69], then providing community-based support options/interventions for the parents of these children may be important for preventing the detrimental effects of stress on family functioning and well-being.

The higher incidence of hyperactivity-inattention problems in children with mild-to-moderate autistic traits, observed here, accords with the finding that many children with ASD have comorbid ADHD symptoms [70]. Likewise, ADHD symptoms have been associated with mild-to-high autistic traits in school-aged children and adults [7]. The present findings showed this association but at a younger age, indicating the early manifestation of an association between autistic traits and ADHD symptoms. Although the mechanisms underlying the association between autistic traits and ADHD symptoms are uncertain, evidence from several twin studies indicates a substantial genetic influence [7, 71].

Another notable finding is that peer problems were associated with the highest odds in both the Medium- and High-risk groups (Wave 1). This finding accords with the results from an earlier population-based study, which showed that autistic traits in children and adolescents measured by the SRS were associated with negative peer relationships and problematic peer interactions [72]. There are various factors that might underlie this association. For example, conduct problems and hyperactivity are known to be associated with peer rejection in children aged 3–5 years [73] and as previously mentioned, our study found that children in the Medium−/High-risk groups had greater conduct problems and higher hyperactivity scores. In addition, factors intrinsic to autism itself, such as social communication difficulties, that were elevated in both the Medium- and High-risk groups, might also be important in this context. As the preschool period marks the initial phase where children can develop competency in interacting with peers, our results highlight the importance of detecting autistic traits in young children to prevent their potentially detrimental impact on early social development.

Based on the diagnostic assessment, 25 children who were categorized as having mild-to-high autistic traits were not diagnosed as having ASD. Importantly, the assessments allowed us to not only obtain diagnostic information on autistic traits, but also, information on overall mental health including the children’s intellectual ability. We found that besides increased emotional/behavioral problems, children with subthreshold autistic traits also had lower cognitive performance as measured by the WPPSI. This result is consistent with those from previous studies that have shown impaired cognitive abilities in children/young adults with subthreshold autistic traits [12, 60, 74]. In particular, Möricke et al. (2010) reported lower performance in language comprehension and production tasks in children with subthreshold autistic traits at age 4–5 years [12]. In addition, a population-based study that examined the association between nonverbal IQ and autistic traits in children aged 6- to 10-years old [60] with subthreshold autistic traits found a relationship between autistic traits and cognitive functioning even after adjusting for sociodemographic factors. The results from these studies in conjunction with our findings suggest that subthreshold autistic traits may relate to lower performance in various aspects of cognitive functioning including attention, executive functioning, memory and learning, language, and visuospatial functioning, which in turn, might also be important for the emotional and behavioral problems children with subthreshold autistic traits experience in the preschool period. Indeed, even though there has been little research on this to date, a recent study that used a clinical sample showed that although 4- to 8-year old children with ASD had higher emotional/behavioral problems regardless of their intellectual ability, children with an IQ below 70 had higher self-absorption and hyperactivity scores while children with an IQ above 70 had increased disruptive behavior, depression, and anxiety symptoms [75]. These findings suggest that comorbid cognitive difficulties might be important in understanding the specific etiology of emotional and behavioral problems in autistic children and possibly children with increased autistic symptomatology.

### Limitations

This study has several limitations. First, the data used in this study were cross-sectional which did not allow us to fully explore the associations in terms of their temporal ordering. As research on other negative outcomes (internalizing problems) linked to autistic traits in childhood and early adolescence has indicated that there may be an asymmetric bidirectional association [66], it highlights the importance of studying these associations across time in order to better delineate them. As one of the aims of the TCS is to clarify the cross-sectional relationship between autistic traits and co-occurring mental health problems as well as the developmental trajectories of autistic traits and co-occurring mental health problems, the results from the current study underscore that subthreshold autistic traits are associated with emotional/behavioral problems and cognitive difficulties in an analogous fashion to the way these outcomes are observed in children with ASD. Future research is necessary to clarify that these associations are maintained across time and determine whether different levels of autistic traits have different trajectories in their associations with mental health problems.

Second, as information on ASD symptoms in Wave 1 was obtained from caregivers’ reports and not obtained in a full diagnostic interview, we cannot rule out the possibility of rater effects in the observed associations. Third, although categorized as discrete disorders in DSM-5, ASD and some psychiatric disorders such as obsessive-compulsive disorder (OCD) or developmental coordination disorder (DCD) share some characteristics. For example, peer difficulties and social communication difficulties have been reported in individuals with DCD [76–78]. Therefore, we cannot discount the possibility that other conditions might have played a role in the observed associations. However, autistic traits are assumed as a common vulnerability factor for various mental disorders as mentioned earlier [23, 79]. It is assumed that assessing subthreshold autistic traits in preschool is important for the early detection of high-risk children for various mental disorders. Fourth, while we were able to undertake a diagnostic assessment, which is a strength of this study, the modest sample size and high non-participation rate of randomly/non-randomly invited families may have affected the results. Finally, because of the small number of children that took part in the diagnostic assessment, we were not able to more precisely explore the role of cognitive factors in the association between subthreshold autistic traits and emotional and behavioral problems.

## Conclusion

This study showed that subthreshold autistic traits are associated with an increased risk of emotional/behavioral problems and lower cognitive functioning in preschool children. As the influence of autistic traits in the etiology of emotional/behavioral problems in young children may be missed in the absence of a formal diagnosis, the results of this study highlight the importance of raising awareness among clinicians and educators that even subthreshold autistic traits may increase the risk for psychosocial and cognitive difficulties that can potentially impede children’s social development. Our findings suggest that it might be efficacious to consider autistic traits as a possible risk factor for emotional/behavioral problems and lower cognitive performance in early childhood and facilitate screening for them, especially when parents have concerns about their children, even at non-specialized primary health care services. Follow-up assessments of these children can detect any potential problems as early as possible and provide them with the necessary support.

## Data Availability

The datasets generated and analyzed during the current study are not publicly available due to the need to protect children’s personal information but are available from the corresponding author upon reasonable request.

## Abbreviations

ADHD: Attention deficit hyperactivity disorder
ADOS: Autism Diagnostic Observation Schedule
ADI-R: Autism Diagnostic Interview-Revised
ASD: Autism spectrum disorder
CBCL: Child Behavior Checklist
DCD: Developmental coordination disorder
DSM: Diagnostic and Statistical Manual of Mental Disorders
IQ: Intelligence quotient
OCD: Obsessive compulsive disorder
SRS: The Social Responsiveness Scale
SDQ: The Strengths and Difficulties Questionnaire
TCS: The Tama Children’s Survey
TQI: The case-finding instrument tool
WPPSI: Wechsler Preschool and Primary Scale of Intelligence
